# Detection of Glycosylated Markers From Cancer Stem Cells With ColoSTEM Dx Kit for Earlier Prediction of Colon Cancer Aggressiveness

**DOI:** 10.3389/fonc.2022.918702

**Published:** 2022-07-22

**Authors:** Sabrina Blondy, Stéphanie Durand, Aurélie Lacroix, Niki Christou, Charline Bouchaud, Maud Peyny, Serge Battu, Alain Chauvanel, Vincent Carré, Marie-Odile Jauberteau, Fabrice Lalloué, Muriel Mathonnet

**Affiliations:** ^1^ Carcidiag Biotechnologies company, Guéret, France; ^2^ INSERM U1308 - CAPTuR “Control of cell activation, Tumor progression and Therapeutic resistance”, Faculty of Medicine, University of Limoges, Limoges, France; ^3^ Department of Digestive Surgery, Dupuytren University Hospital, Limoges, France; ^4^ Laboratory of Analytical Chemistry, Faculty of Pharmacy, Limoges, France; ^5^ Department of Pathology, Dupuytren University Hospital, Limoges, France; ^6^ Department of Immunology, Dupuytren University Hospital, Limoges, France

**Keywords:** colorectal carcinoma, early stage, cancer stem cells, glycosylated biomarkers, prognosis value, tumor aggressiveness

## Abstract

Nowadays, colon cancer prognosis still difficult to predict, especially in the early stages. Recurrences remain elevated, even in the early stages after curative surgery. Carcidiag Biotechnologies has developed an immunohistochemistry (IHC) kit called ColoSTEM Dx, based on a MIX of biotinylated plant lectins that specifically detects colon cancer stem cells (CSCs) through glycan patterns that they specifically (over)express. A retrospective clinical study was carried out on tumor tissues from 208 non-chemotherapeutic-treated and 21 chemotherapeutic-treated patients with colon cancer, which were stained by IHC with the MIX. Clinical performances of the kit were determined, and prognostic and predictive values were evaluated. With 78.3% and 70.6% of diagnostic sensitivity and specificity respectively, our kit shows great clinical performances. Moreover, patient prognosis is significantly poorer when the MIX staining is “High” compared to “Low”, especially at 5-years of overall survival and for early stages. The ColoSTEM Dx kit allows an earlier and a more precise determination of patients’ outcome. Thus, it affords an innovating clinical tool for predicting tumor aggressiveness earlier and determining prognosis value regarding therapeutic response in colon cancer patients.

## Introduction

Colon cancer represents the second leading cause of death from cancer (1–3). Diagnosis is usually based on the pathological staging classification (pTNM) (stages I to IV) (4). Surgical resection is the only curative method at present. Although the prognosis has improved in recent years, survival rates widely vary by stage, with 85% 5-years net survival for stage I and 50% for stage III (5). Indeed, nearly 10% of stage I, 30% of stage II and 55% of stage III will present a metachronous cancer or a recurrence (locoregional or metastatic) within 5 years postoperatively (6). This high risk of relapse requires to further improve earlier detection of colon cancer and to achieve personalized evaluation of patients’ outcome and prognosis. This approach implies a systematic and precise determination of disease aggressiveness in order to strengthen patient follow-up and management (7). Therefore, searching and using for molecular biomarkers pave the way to improve patients’ prognosis and management. Since abnormal glycosylation is a common phenomenon that occurs in cancer cells (8), thus, glycan abnormalities profiles play important roles in cancer biology and therefore afford a potential tool for the characterization of tumor markers.

Glycosylation is one of the most important posttranslational modifications of lipids (glycolipids) and proteins (glycoproteins), by the highly coordinated action of glycosyltransferases and glycosidases. Glycoproteins and glycolipids regulate a diverse range of key biological and cellular functions, including differentiation, proliferation, growth, pluripotency *etc …* Alterations in glycosylation processes (*i.e.* aberrant glycosylation) are linked to colon cancer development, progression, metastases and therapeutic failures (9–11). Aberrant glycosylation constitutes a hallmark of Cancers and might even lead to the acquisition of a stemness phenotype (12–15). This mechanism is already known to be altered in proteins expressed on tumor differentiated cells but also in a specific cell subpopulation with acquired stemness properties, the cancer stem cells.

Cancer Stem Cells (CSCs) are highly tumorigenic cells, *i.e.*, they are able to give rise to complete tumor mass. In addition to their ability to regenerate tumor mass, the main CSCs properties are self-renewal and multipotent differentiation capacity. They also have a unique property of resistance to treatments. Given all these features, CSCs contribute to colon tumor initiation, progression (metastasis formation), aggressiveness and relapses (16–21) and represent new biomarkers for cancer prognosis due to their original stemness properties. Various CSCs markers were defined to identify and isolate colon CSCs. Most of them are (i) membrane receptors and surface molecules (such as CD44 and its splice variants (CD44v), CD133/1 (AC133 epitope) or epithelial cell adhesion molecule (EpCAM)), (ii) cytosolic enzyme, such as aldehyde dehydrogenase 1 (ALDH1) enzymatic activity, and transcription factors including OCT-4 (12, 16, 17, 19, 22, 23). However, these biomarkers currently failed to be used in clinic because of their concomitant expression in non-tumor stem cells (SCs) (12, 13). These data underline the importance of evidence of more specific colon CSCs biomarkers.

Some data reported a correlation between the alteration of glycosylation processes with the induction and/or regulation of CSCs phenotype and properties. Recent results evidenced that colon cancer stemness would be regulated by O-GlcNAcylation. Indeed, the inhibition of the O-GlcNAc transferase (OGT) and thus of the GlcNAc residue leads to an increase of colon cancer stemness characteristics and properties, concomitant with a more aggressive and malignant phenotype (24). Another study evidenced that overexpression of O-glycan truncated forms such as Tn antigen (Ag), is involved in the development and the induction of colon oncogenic features (tumorigenesis, cell growth, invasion, metastases and resistance to UV-induced apoptosis) (25). The expression of ß-1,4-N-acetylgalactosaminyltransferase 3 is upregulated in colonospheres and its knockdown decreases sphere formation and stemness marker expression (OCT-4 and NANOG) (26). Overexpression of α-2, 6-Sialyltransferase and α-N-acetylgalactosaminide α-2,6-sialyltransferase 1, are both correlated with (i) colon CSCs enrichment (increase of CD133 and ALDH1 expressions, as well as sphere forming ability), and (ii) acquired resistance to chemotherapy (irinotecan and 5-Fluorouracil) and EGFR-targeted therapy (gefitinib) (27–30). *FUT9* gene encoding the α-1,3 fucosyltransferase, plays a complex dual role in colon cancer development and malignancy. Alpha-1,3 fucosyltransferase knockdown strongly decreases sphere formation, growth of xenograft tumors and expression of OCT-4 and CD44, whereas it increases cell proliferation and migration. *FUT9* expression supports colon cancer aggressiveness. Its expression at early stages is required for CSCs expansion and colon cancer initiation. On the contrary, its downregulation at later stages promotes colon cancer progression (31). Most colon CSCs surface markers are glycoproteins. They differ from their normal counterpart by the expression of tumor specific glycans (15). Thus, CD44 splice variants carry oncofetal carbohydrate T and sialyl-Tn (sTn) Ag, correlating with the increased metastatic potential of colon cancer cells. The case of CD133 can also be mentioned since rather than the expression of total CD133 protein, it is the expression of a specific glycan epitope (AC133) that could constitute a “bona fide” CSCs marker. Altogether these data suggest that a better characterization of colon CSCs glycosylation profiles could pave the way to identify more efficient new CSCs biomarkers in order to improve specific detection within tumor and thus for targeting them.

Based on these knowledges and current clinical needs, Carcidiag Biotechnologies company has developed the ColoSTEM Dx kit, consisting of specific colon CSCs detection within heterogeneous tumor cell populations. There is currently no clinically standardized way (*i.e.*, efficient prognosis biomarkers) to provide a reliable and earlier prognosis for colon cancer patients. In this context, our kit provides innovating and reliable biomarkers, specific to colon CSCs, for a better and an earlier stratification of low- or high-risk patients to develop an aggressive disease and relapse. The ColoSTEM Dx kit represents a tool perfectly adapted to the personalized management of patients. More precisely, it is an innovative tool that uses a MIX of biotinylated plant lectins (UEA-1, Jacalin and ACA, mixed in a particular *ratio*) recognizing glycan patterns specifically (over)expressed by colon CSCs and tumor cells related to CSCs. These glycan patterns are not expressed neither by “normal” stem cells nor by differentiated cancer tumor cells. This colon CSCs specific MIX was evidenced by lectin-arrays and validated *in vitro* from research works carried out in collaboration with the University of Limoges, that have conducted to file two patents (national registration numbers WO2016FR53196 and WO2016FR53197).

Based on these results and in collaboration with Limoges University Hospital, we conducted a retrospective clinical study aiming at validating the ColoSTEM Dx kit for a routine clinical use by immunohistochemistry (IHC).

## Materials and Methods

### Cell Culture

Colon adenocarcinoma cancer cell lines HT-29 were obtained from ATCC (HTB-38™; ATCC^®^, France). Cells were incubated at 37°C with 5% CO2 and 95% humidity and cultured in 1X McCoy’s 5A modified medium (Gibco - ThermoFisher Scientific, France) supplemented with 10% fetal bovine serum (FBS; Gibco- ThermoFisher Scientific, France) and 1% penicillin/streptomycin (Gibco - ThermoFisher Scientific, France).

### Indirect Magnetic Cell Sorting

MACS was realized from 10^7^ cells using the CELLection™ Biotin Binder kit (Invitrogen - ThermoFisher Scientific, France) according to manufacturer’s instructions, using 10µg of (i) the MIX (ColoSTEM Dx kit) or (ii) the AC133 biotinylated antibody (Ab) (Miltenyi Biotech, France), were used.

### Evaluation of EpCAM^High^ Immunostaining and ALDH1^bright^ Activity by Flow Cytometry

EpCAM^high^ cell percentages within the AC133 – and + sorted-cells, were analyzed by flow cytometry (FCM) from 5.10^4^ cells. After saturation in 1% BSA in DPBS 1X calcium and magnesium free (10min, 4°C), cells were incubated for 45min at room temperature (RT) with an EpCAM mouse monoclonal Ab (clone VU1D9; Ozyme - Cell Signaling Technology, France) diluted at 1:150 in 1% BSA/DPBS. After a washing step in DPBS 1X (g x 300, 10min, 4°C), cells were incubated for 30min at RT in the dark with an Alexa-Fluor 633-conjugated goat anti-mouse secondary Ab (ThermoFisher Scientific, France) diluted at 1:1000 in DPBS 1X.

Enzymatic activity of ALDH1 in MIX+ and MIX- sorted-cells was analyzed from 10^5^ cell/mL, using the ALDEFLUOR kit (Stem Cell Technologies, France) according to the manufacturer’s recommendations.

Cells were extemporaneously stained with a DNA dye, i.e., 0.5µL propidium iodide (PI; λex=475-581nm/λem=583-697nm; BD Biosciences, France). EpCAM^high^ immunostaining and ALDH1 enzymatic activity (ALDH1bright cells) were analyzed among live cells (PI-), with the BD AccuriC6 Plus FCM (BD Biosciences, France). Mouse IgG1 isotype control (R&D systems, France) and N, N-diethylaminobenzaldehyde (DEAB, ALDH1 specific inhibitor) were used to control for background fluorescence

### Clonogenicity Assay

MIX- and MIX+ sorted-cells were seeded in ultra-low attachment 96-wells plates (Falcon Corning brand, France) in increasing cell densities, i.e., 600, 1250, 2500, 5000, 10000 cells. For each condition, cells were seeded in 3 wells in 200µL of defined medium composed of 1X Dulbecco’s Modified Eagle’s Medium (DMEM)/F12 (HAM 1:1) medium (Gibco - ThermoFisher Scientific, France) supplemented in 1X B27/Insulin (ThermoFisher Scientific, France), 10ng/mL Fibroblast Growth Factor (FGF; Peprotech, France) and 20ng/mL Epidermal Growth Factor (EGF; Peprotech, France). Twenty microliters of medium were added per well every week, for 4 weeks. The number of spheres formed per well and per condition was counted under optical microscope (Olympus CKX53; magnification, x100).

### Patients and Samples

Ninety colon tumor tissues came from TMA (Tissue MicroArray) (HCol-Ade180Sur-08; AMSBIO, USA), which also includes the 90 corresponding healthy borders. Necrosed and absent tumor tissues (N=4) were excluded (N=86).

Forty-six colon tumor tissues were collected from patients having benefited from colon cancer resection at the Department of Digestive Surgery, General and Endocrine Surgery at Limoges University Hospital (France) and without any pre-operative chemotherapeutic treatment. Necrosed (N=4) were excluded (N=42).

Twenty-four tumor tissues were also collected from colon cancer patients with pre-operative chemotherapy at Limoges University Hospital (France). Necrosed (N=3) were excluded (N=21).

Supplementary colon tumor tissues were collected from two TMA constituted in a cohort of colon cancer patients with early stages (I and II) (without chemotherapeutic treatment) from the “Centre de Ressources Biologiques – Institut Régional du Cancer Montpellier (CRB-ICM, Montpellier, France, ICM-CORT-2016-26). Necrosed and absent or non-interpretable tumor tissues (N=15) were excluded (N=80). Both TMAs also include N=50 paired healthy samples.

Eighteen kidney tumors (clear cell carcinoma) in TMA were chosen as MIX negative control provided from AMSBIO society and are referenced as T8235714D-5 and T8235716D-5.

Clinicopathological data including pTNM stages, gender, age and survival status at 5- and 7-years were provided after baseline examinations and patients’ diagnosis according to histological analyses of biopsies (American Joint Committee on Cancer staging manual) (32). Survival rates analysis of non-chemotherapeutic-treated and chemotherapeutic-treated patients from all stages (refer to “statistical analysis” described below) were realized respectively from N=128 and N=21 samples (**Tables S1** and **S2**). Survival rates analysis at 5 years of non-chemotherapeutic-treated patients from early stages (refer to “statistical analysis” described below) were realized from N=70 samples (N=29 from CRB-ICM, N=27 from Limoges Hospital and N=14 from the TMA (AMSBIO) (**Table S3**).

### MIX and OCT-4 IHC Immunostaining

MIX staining was realized on N=208 and N=21 tumor tissues from respectively non-chemotherapeutic-treated and chemotherapeutic-treated patients with colon cancer (refer to “patients and samples” section; **Tables S1**, **S2** and **S3**). MIX/OCT-4 co-staining was performed on some tumor tissues from the non-chemotherapeutic-treated patients’ cohort, i.e., N=42 tumor tissues from Limoges University Hospital (refer to “*Patients and Samples*” section; **Table S1**). MIX staining was also achieved on 18 kidney tumors samples in order to assess sensibility and specificity in comparison with MIX staining performed in colon cancer samples. Each staining was realized by IHC on paraffin-embedded histological sections (4μm in thickness), in three main steps using the Leica Bond Max automatic staining platform (Leica Biosystems, France), according to the manufacturer’s instructions: (i) Preparation and pretreatment of the tissues. Paraffin coating is removed using the Bond Dewax Solution (Leica Biosystems, France) and tissues are rehydrated under heat using the acidic buffer Bond Epitope Retrieval Solution 1, for 5min (pH 6; Leica Biosystems, France); (ii) Immunostaining. Activity of endogenous peroxidases and biotins was blocked using the Bond Intense R Detection kit (Leica Biosystems, France). Tissues were incubated for 20 min with either the MIX alone pre-diluted at 1:2 ratio in the diluent supplied in the ColoSTEM Dx kit, or with both MIX (1:2) and OCT-4 (OCT-4 polyclonal rabbit IgG Ab; ThermoFisher Scientific, France). MIX and/or OCT-4 staining was revealed using respectively the Bond Intense R Detection kit and the Bond Polymer Refine Red Detection kit (Leica Biosystems, France). Nucleus were counter-stained by incubation with hematoxylin (Leica Biosystems, France) for 8 min; (iii) Slides mounting. After dehydration by two successive baths of absolute ethanol (VWR, France) and toluene (ThermoFisher Scientific, France), for 5min each, tissue slides were mounted using the Leica CV Ultra (Leica Biosystems, France) and examined under the Leica photomicroscope DM4 B (Leica Biosystems, France; 200x magnification).

### Scoring Method

**Figure 1:**
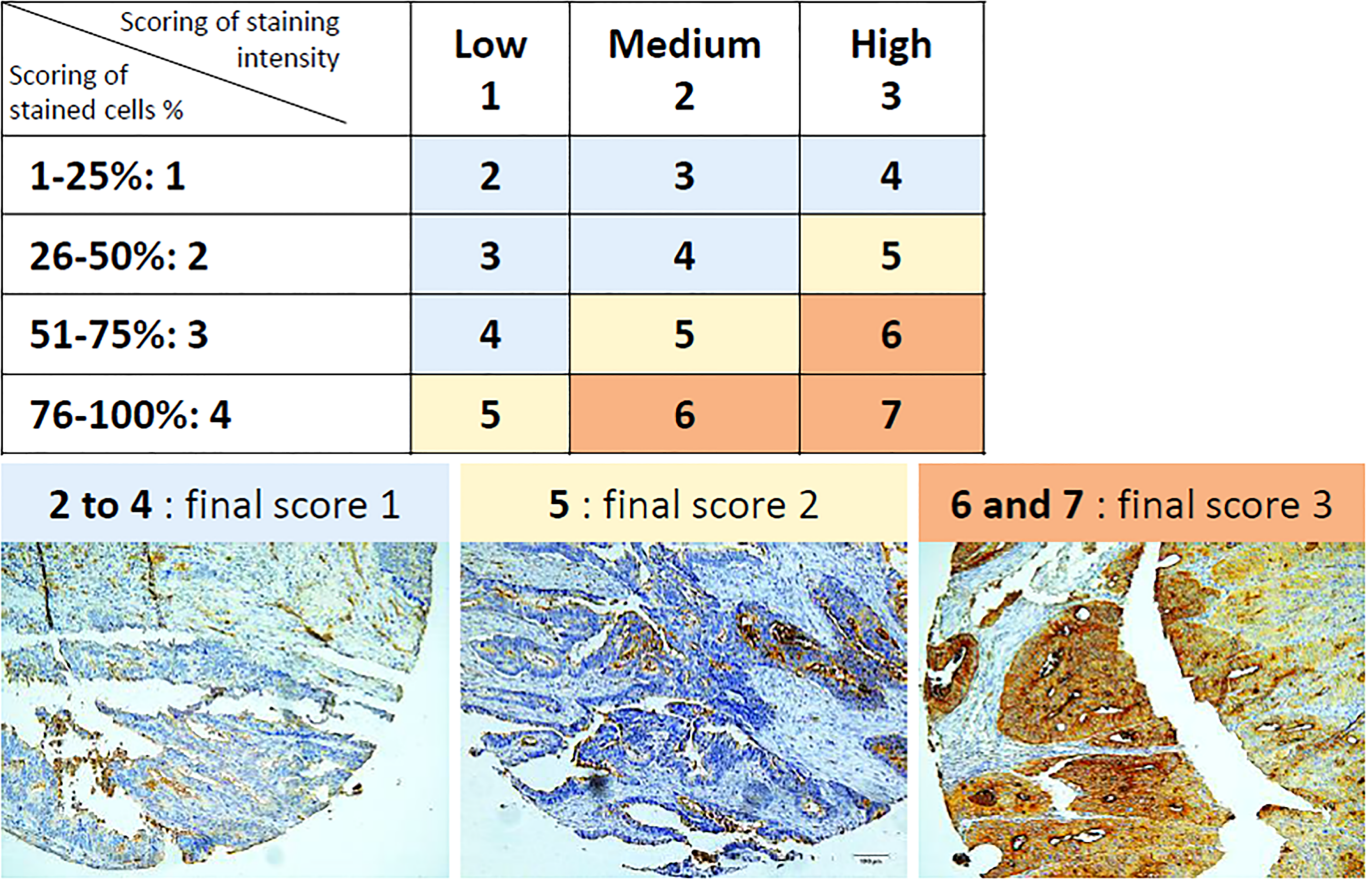
Scoring method of IHC staining, according to percentages of stained cells and staining intensity. Percentages of stained cells are graduated into four scores (1 to 4) and staining intensity is graduated into three scores (1 to 3). The total some of both scoring results in intermediate scores ranging from 2 to 7. Intermediate scores ranging from 2 to 4 (in blue) result in a final score of 1. Intermediate score of 5 (in yellow) results in a final score of 2. Intermediate scores of 6 and 7 (in orange) result in a final score of 3. Final scores of 1 and 2 are considered as “Low staining” and final score of 3 is considered as “High staining”. Representative illustrations of MIX staining (in brown), as observed by IHC (magnification, 200x), are depicted below table. *IHC: Immunohistochemistry*.

MIX staining appears in brown at apical membrane and/or in cytoplasm. OCT-4 staining appears in red/pink within nucleus (in blue) and/or cytoplasm. Scoring method of both staining was adapted from a previous an well-known scoring method called “quickscore” (33–35). All tissues were stained either with the MIX alone, or with both the MIX and OCT-4. The total absence of staining (score 0) or the presence of stained cells constitutes the first element of analysis. The second element of analysis is related to the proportion of stained cells that is scored according to the followed gradation: 1 = 1-25%, 2 = 26-50%, 3 = 51-75% and 4 = 76-100% of stained cells. The third element of analysis is related to the staining intensity, graduated as followed: 1 = Low, 2 = Medium and 3 = High staining intensity. Scores obtained from both gradations are then added together and the total obtained results into 6 intermediate scores, ranging from 2 to 7, which are finally grouped into 3 final scores (**Figure 1**). Final scores of 1 and 2 are considered as “Low staining” (MIX-Low and/or OCT-4-Low) and final score of 3 is considered as “High staining” (MIX-High and/or OCT-4-High).

### Evaluation of Clinical Performances of the ColoSTEM Dx Kit

Clinical performances of the ColoSTEM Dx kit, were determined from N=166 tumor tissues (N=86 of the commercial TMA (AMSBIO) and N=80 of the cohort of CRB-ICM Montpellier) and N=136 non-tumor tissues (N=86 tumor borders of the commercial TMA (AMSBIO) and N=50 non-tumor samples of the cohort of CRB-ICM Montpellier). Diagnostic sensitivity is related to the percentages of tumor tissues stained with the MIX (true positives) relative to the unlabeled ones (false negatives), as followed Sensitivity (%) = 100 x (True positives/(True positives + False negatives)). Diagnostic specificity is related to the percentages of tumor borders or non-tumor tissues unstained with the MIX (true negatives) relative to the labeled ones (false positives), as followed Specificity (%) = 100 x [True negatives/(True negatives + False positives)].

### Statistical Analysis

Statistical analysis and graphics were performed with StatView 5.0 (USA), Prism 7 (GraphPad, USA) and R environment (version 4.0.3). Statistical analysis of *in vitro* clonogenicity assay was made with an ANOVA/ANCOVA test. Survival rates according to MIX/OCT-4 co-staining were analyzed from the non-treated patients from the Limoges Hospital cohort, at 5 years, *i.e*., only patients whose survival is ≤ 60 months at the last visit time, were retained (N=42; refer to “patients and samples” and **Table S1**). Survival rates of all non-treated patients were analyzed according to their clinicopathological data and MIX staining at (i) 5 years and (ii) 7 years, i.e., patients whose survival is ≤ 84 months at the last visit time (respectively N=79 and N=128; refer to “patients and samples” and **Table S1**). Survival rates analysis at 5 years of non-treated patients from early stages were achieved by combining three cohorts composed of patients from Limoges’ Hospital, CRB-ICM Montpellier as well as from a cohort provided by AMSBIO (N=70; refer to “patients and samples” and **Table S3**). Survival rates of treated patients were analyzed according to MIX staining, at 5 years (N=21; refer to “patients and samples” and **Table S2**). The prognostic value of each parameter for outcome was assessed using the Kaplan-Meier method and log-rank test (Mantel-Cox). For each variable, hazard ratio (HR) was estimated using a univariate Cox model and expressed with their 95% confidence interval (95% CI). Multivariate analysis was carried out using a Cox regression model on single features identified by the univariate Cox modeling. Survival analysis were performed in R using *survival* and *survminer* packages. The proportional hazards assumption for Cox regression model fit was verified using *cox.zph* function of survival package. A p-value below 0.05 was considered as significant.

## Results

### The ColoSTEM Dx Kit Allows Efficient Isolation and Enrichment of a Cell Subpopulation With Stemness Properties

The ColoSTEM Dx kit originates from research works carried out by the EA3842 laboratory (Limoges’ University; patent national registration number 1561763 – publication number 3044680). It aims at colon CSCs specific detection in both heterogeneous colon cancer cell populations and tumors. Indeed, it is based on the use a MIX of biotinylated plant lectins that recognize glycan patterns specifically (over)expressed by these cells, *i.e*., not by differentiated cancer cells, within heterogeneous tumor colon tissues. ColoSTEM Dx kit proofs of concept, *i.e*., MIX evidence by lectin-arrays and its validation in specific colon CSCs detection and enrichment from several colon adenocarcinoma cell lines (including HT-29), are reported in detail in the patent mentioned above.

Main and most relevant results have been recalled in **Figure S1**. Briefly, HT-29 cells were sorted by MACS, with either the MIX (MIX+ and MIX- sorted-cells) or an AC133 Ab (AC133+ and AC133- sorted-cells). Some of CSCs characteristics and properties were then evaluated: protein expression and enzymatic activity of stem cells (SCs) markers (EpCAM and ALDH1), and sphere forming ability. While there are as many EpCAM^High^ cells in AC133+ sorted-cells as in AC133- (normalized to 1), there are 7.5-times more EpCAM^High^ cells in MIX+ sorted-cells, compared to both MIX- (normalized to 1) and AC133+ cells (**Figure S1A**). Consistently, there are 4.7-times more ALDH1^bright^ cells in MIX+ sorted-cells (74.73%) than in MIX- (15.6%) (**Figure S1B**). Finally, MIX+ sorted-cells have a significant capacity to form spheres compared to MIX- cells, even when seeded at low densities (**Figure S1C**).

The use of the ColoSTEM Dx kit is more efficient for detecting and enriching in specific colon CSCs than cell sorting using AC133. These results suggest that ColoSTEM Dx kit improves CSCs detection and cell sorting.

### The ColoSTEM Dx Kit Improves Colon CSCs Detection and Allows a More Accurate Prognosis Than the Standard Stem Cell Marker OCT-4

MIX specificity in colon CSCs detection by IHC on tumor tissue, as well as its efficiency in patients’ prognosis evaluation, were evaluated and compared to a standard SCs marker, OCT-4. MIX and OCT-4 staining were realized on N=42 tumor tissues from non-chemotherapeutic-treated patients (**Table S1** and **Figure S2**). Among stained samples, half of samples are MIX-Low or MIX-High (**Figure S2A**), while OCT-4-high staining is present in a broad panel of samples (almost 80%, **Figure S2B**), suggesting that OCT-4 is not able to discriminate CSCs from the heterogeneous cell subpopulations. In addition, when cells are double-stained with MIX and OCT-4, samples are mainly divided in MIX-Low/OCT-4-High or MIX-High/OCT-4-High (**Figure S2C**). Intensity of MIX staining is not linked to OCT-4 staining and is independent of clinicopathological characteristics of patients except for gender (**Table 1**). However, this association is not found later on larger patient cohorts (**Table 2**). Altogether, these results suggest that the ColoSTEM Dx kit is relevant for the discrimination of cancerous from healthy SCs. It also evidences a better specificity to detect colon CSCs, than OCT-4 whose staining within tumor colon epithelium does not seem to be restricted to CSCs, but to all SCs (healthy and cancerous) and progenitors.

**Table 1:** Evaluation of the relationship between each clinicopathological characteristic (Sex, Age and pTNM staging) of non-chemotherapeutic-treated patients (n = 41, **Table S1**) and intensity of MIX staining (Low/High) by Chi-squared statistic.

		n	MIX staining		
			Low	High	P value
**Sex**	**Female**	18	12	6	**0.046**
	**Male**	23	7	16	
**Age**	**< 60yrs**	4	2	2	1
	**≥ 60yrs**	37	17	20	
**Stage (UICC)**	**Early (I/II)**	27	14	13	0.514
	**Late (III/IV)**	14	5	9	
**OCT4 staining**	**Low**	8	6	2	0.109
	**High**	33	13	20	

Association between scoring according to MIX and OCT-4 staining has been evaluated. pTNM, pathology Tumor-Node-Metastasis, UICC, Union for International Cancer Control; yrs, years.

The p values less than or equal to 0.05 have been written in bold.

**Table 2:** Relationship between intensity of MIX staining and clinicopathological characteristics of non-treated patients included at 5 years and 7 years of OS.

(A)					
		n	MIX Staining		
			Low	High	P value
**Sex**	**Female**	35	18	17	0.239
	**Male**	38	14	24	
**Age**	**< 60yrs**	8	6	2	0.139
	**≥ 60yrs**	65	26	39	
**Stage (UICC)**	**Early (I/II)**	41	17	24	0.809
	**Late (III/IV)**	32	15	17	
(B)					
		**n**	**MIX Staining**		
			**Low**	**High**	**P value**
**Sex**	**Female**	54	22	32	0.855
	**Male**	61	23	38	
**Age**	**< 60yrs**	16	11	5	**0.014**
	**≥ 60yrs**	99	34	65	
**Stage (UICC)**	**Early (I/II)**	73	24	49	0.087
	**Late (III/IV)**	42	21	21	

Numbers of tumor tissues from non-treated patients included at 5 years (A) and 7 years (B) of OS, for which there is a MIX-Low staining or a MIX-High staining, according to clinical and pathological data, i.e., gender, age and stage, were indicated. The association between MIX staining and clinicopathological characteristics of patients was evaluated by Chi-squared statistic. **(A)** All tissues (n = 79) were stained with the MIX but 6 samples show absence of MIX staining (not reported in the present table). They are distributed as follows: gender: 3 Female/3 Male; age: 3 < 60 yrs/3 ≥ 60 yrs; stage: 3 early/3 late; vital status: 1 alive/5 dead. (B) All tissues (n = 128) were stained with the MIX but 13 samples show absence of MIX staining (not reported in the present table). They are distributed as follows: gender: 6 Female/7 Male; age: 4 < 60 yrs/9 ≥ 60 yrs; stage: 7 early/6 late; vital status: 7 alive/6 dead. OS, Overall Survival; UICC, Union for International Cancer Control; yrs, years.

The p values less than or equal to 0.05 have been written in bold.

**Figure 2:**
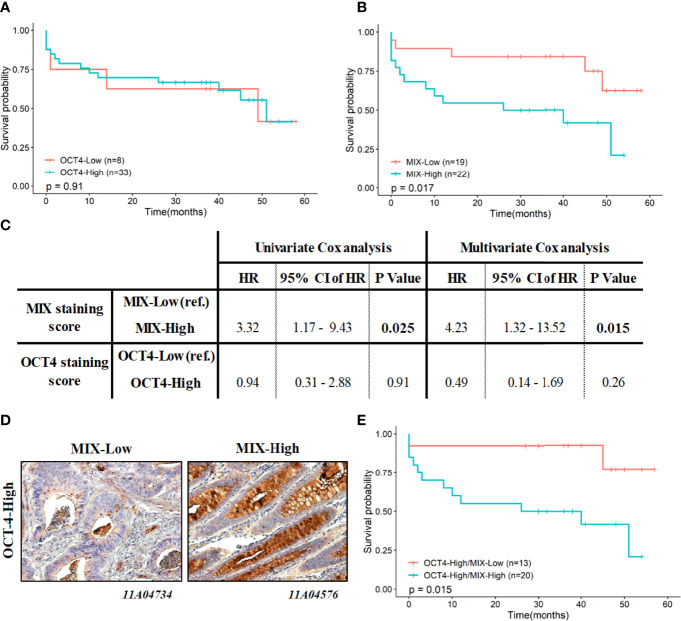
Association between MIX and OCT-4 scoring with survival rates at 5 years (60 months). Kaplan-Meier curves at 5-years are depicted according to OCT-4-Low *versus* -High staining **(A)**, MIX-Low *versus* -High staining **(B)** and MIX-High/OCT-4-High *versus* MIX-Low/OCT-4-High co-staining **(E)**. P values indicated in each panel are related to the log-rank tests (Mantel-Cox) performed to survival curves comparison. **(C)** Prognostic value of MIX and OCT-4 scoring independently, was estimated using univariate and multivariate Cox regression models, and expressed with their HR and 95% CI. **(D)** Representative illustrations of MIX (brown) and OCT-4 (red/pink) staining, as observed by IHC, are depicted (magnification, 200x)*. CI: Confidence Interval; HR: Hazard ratio; IHC: Immunohistochemistry*.

Survival rates at 5 years were evaluated by Kaplan-Meier curves according to either OCT-4 staining (OCT-4-Low *versus* High; **Figure 2A**), MIX staining (MIX-Low *versus* High; **Figure 2B**) or both staining (OCT-4-High/MIX-Low *versus* OCT-4-High/MIX-High; **Figure 2E**). Univariate and multivariate Cox regression were performed to estimate prognosis values and risk scores associated to OCT-4 and MIX staining (**Figure 2C**). Representative pictures of MIX/OCT-4 co-staining, are depicted in **Figure 2D**.

Survival rates at 5-years are not significantly different regardless of OCT-4- staining (Low *vs* High; **Figure 2A**; p=0.91). On the contrary, 5-years survival rates of the MIX-High subgroup are significantly poorer than of the MIX-Low (**Figure 2B**; p=0.017). No relevant difference in survival median having been noted between a MIX-Low and an OCT-4-Low staining (data not shown; p=0.28). Interestingly, even if results are not significant, it has been observed a lower survival median (decrease of 18 months) with a MIX-High compared to an OCT-4-High staining (data not shown; p=0.2). Cox univariate analysis indicated that only MIX staining is a predictive factor for OS, with a significantly increasing risk associated to a MIX-High staining (HR: 3.3, 95% CI 1.17 to 9.43, p=0.025; **Figure 2C**, left panel). Multivariate model confirms independence and relevance of MIX staining as prognostic factor (HR: 4.2, 95% CI 1.3 to 13.5, p=0.015; **Figure 2C**, right panel).

Finally, survival rates at 5 years were also evaluated according to MIX/OCT-4 co-staining on the same tissues (N=41), i.e., MIX-Low/OCT-4-Low, MIX-Low/OCT-4-High, MIX-High/OCT-4-Low, MIX-High/OCT-4-High. Due to not enough MIX-Low/OCT-4-Low and MIX-High/OCT-4-Low tumor tissues included (n=6 and 2, respectively; **Figure S2C**), Kaplan Meier curves were only depicted and analyzed for MIX-Low/OCT-4-High and MIX-High/OCT-4-High co-staining (**Figure 2E**). Interestingly, and consistently with previous observations, a MIX-High/OCT-4-High co-staining predicts significant poorer and worse prognosis than a MIX-Low/OCT-4-High co-staining, with strong survival median decrease (p=0.015; **Figure 2E**). High co-staining harbor a hazard ratio of 5.3 (95% CI 1.2 to 23.8, p=0.0298) in a univariate Cox regression model (not shown).

Contrary to OCT-4, MIX staining levels are closely associated with patient survival. Indeed, compared to OCT-4, MIX-High staining level improve significantly the detection and discrimination of colon CSCs. MIX-High staining might be a relevant CSCs biomarker for monitoring disease aggressiveness and could be useful to establish the prognosis upon treatment.

### The ColoSTEM Dx Kit Allows Earlier Evaluation of Patients’ Disease Aggressiveness and Prognosis, Regardless of Clinicopathological Data

In order to evaluate and confirm the prognosis value of the ColoSTEM Dx kit, all tumor tissues from non-treated patients (N=128, **Table S1**) were stained with the MIX. Survival rates were evaluated at 5- and 7-years of OS, according to clinicopathological data, *i.e*., stages [early (I/II) and late (III/IV)], sex (men and women) and age (< and ≥ 60 years old) (**Table 2**).

Among the 79 tumor tissues included for survival rates analysis at 5-years, 6 were excluded due to a total absence of MIX staining. Fifty-six percent and 44% correspond respectively to early and late stages. Of the 41 early stages, 41% and 59% were respectively MIX-Low and MIX-High. Among the 32 late stages, 47% and 53% were respectively MIX-Low and MIX-High. Regarding OS at 5 years, we noted that MIX-staining is independent of tumor stage (**Table 2A**, p=0.809).

Survival rates at 7 years were then analyzed from 128 tumor samples. As previously described, we excluded tumor samples without MIX staining (n=13). Among the 115 retained tumors, 63% and 37% correspond respectively to early and late stages. Of the 73 early stages, 33% were MIX-Low whereas 67% were MIX-High. Regarding the 42 late stages, half of the population were MIX-Low or MIX-High. This result suggests that regardless of evaluated time point of OS, MIX staining is independent of tumor stages (**Table 2**).

**Figure 3:**
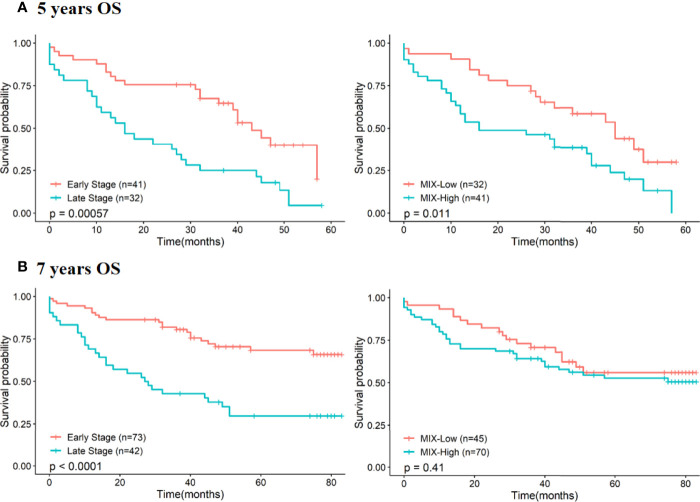
Association between pTNM staging and MIX scoring with survival rates at 5- and 7 years (60 and 84 months respectively). Prognostic value of each feature (pTNM staging and MIX staining) was assessed using the Kaplan-Meier method and log-rank test by stratification of patients according to early and late stages or MIX-Low and High scoring at 5-years of OS **(A)** and at 7-years of OS **(B)**. *OS, Overall Survival; pTNM, pathology Tumor Node Metastasis.*

Kaplan-Meier curves and Cox regression models were displayed and patients’ survival rates were analyzed at 5- and 7-years, according to MIX staining levels and stages (**Figure 3** and **Table 3**). At 5-years, prognostic significance for OS of MIX scoring is clearly supported by survival curve (p=0.011; **Figure 3A**) and univariate Cox model (HR: 2.1 with 95% CI 1.17 to 3.75, p=0.013; **Table 3A**). On the contrary, no statistically relevant difference is shown between MIX-Low or -High at 7-years survival rates (**Figure 3B** and **Table 3B**).

**Table 3:** Prognostic values of clinicopathological features (gender, age, stage and MIX staining) at 5- and 7 years of patients’ follow-ups (60 and 84 months respectively).

(A)										
				Univariate Cox analysis			Multivariate Cox analysis			
				HR	95% CI of HR	P Value	HR	95% CI of HR	P Value
**Sex**		**Female (ref.)**							
		**Male**		1.38	0.78 - 2.44	0.269	/	/	/
**Age**		**< 60 yrs (ref.)**							
		**> 60 yrs**		0.78	0.33 - 1.85	0.573	/	/	/
**Stage (UICC)**		**Early (I/II) (ref.)**							
		**Late (III/IV)**		2.62	1.49 - 4.62	**0.001**	2.997	1.69 - 5.32	**0.0002**
**MIX staining score**		**MIX-Low (ref.)**							
		**MIX-High**		2.09	1.17 - 3.75	**0.013**	2.461	1.37 - 4.44	**0.003**
(B)									
			**Univariate Cox analysis**					**Multivariate Cox analysis**
				HR	**95% CI of HR**	**P Value**	**HR**	**95% CI of HR**	**P Value**
**Sex**		**Female (ref.)**								
		**Male**		1.07	0.62 - 1.85	0.818	/	/	/
**Age**		**< 60 yrs (ref.)**								
		**> 60 yrs**		1.30	0.56 - 3.06	0.542	/	/	/
**Stage (UICC)**		**Early (I/II) (ref.)**								
		**Late (III/IV)**		3.21	1.84 - 5.6	**3.99e-05**	3.77	2.12 - 6.71	**6.51e-06**
**MIX staining score**		**MIX-Low (ref.)**								
		**MIX-High**		1.27	0.72 - 2.26	0.411	1.82	1.00 - 3.30	**0.049**

Resulting HR (with 95% CI), stratifying patients for 5-years of OS **(A)** and for 7-years of OS **(B)** according to clinicopathological features, were obtained by univariate Cox modeling (left panel). Multivariate analysis (right panel) was carried out using a Cox regression model using pTNM staging and MIX scoring. CI, Confidence Interval; HR, Hazard Ratio; OS, Overall Survival; pTNM, pathology Tumor Node Metastasis.

The p values less than or equal to 0.05 have been written in bold.

Noted that sex and age have not a significant impact on survival rates, at both 5- (**Table 3A** and **Figure S3A**) and 7-years (**Table 3B** and **Figure S3B**). Kaplan-Meier curves performed on separately groups of patients (MIX-Low and -High), stratifying according to sex (male and female) or age (inferior or superior to 60 years old), failed to show any difference in survival rates according to low or high MIX staining (data not shown). In brief, no statistically relevant difference on survival rates between MIX-Low or -High staining was noted, regardless of age, even if a significant link was been previously identified by Chi-square test at 7 years patients’ follow-up (p=0.014, **Table 2**). We conclude in the same way with regard to gender.

**Figure 4:**
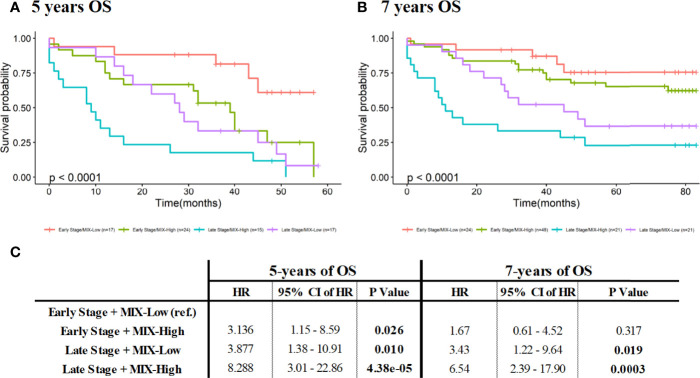
Combination of pTNM staging and MIX scoring for survival analysis at 5- and 7 years (60 and 84 months respectively). Comparison of survival curves was performed using Kaplan-Meier method (with log-rank test) by stratification of patients according to early and late stages or Low- and High-MIX scoring, at 5- **(A)** and 7- **(B)** years. **(C)** Prognostic values of pTNM staging and MIX scoring for survival analysis at 5- and 7 years of patients’ follow-ups were analyzed. Resulting HR (with 95% CI) for stratifying patients for 5- (left) and 7- (right) years of OS using stage and MIX scoring combination, were obtained by univariate Cox modeling. *CI, Confidence Interval; HR, Hazard Ratio; OS, Overall Survival; pTNM, pathology Tumor Node Metastasis*.

Since multivariate analysis revealed that late stages and a high-MIX score were independent prognosis factors at both 5-years and at 7-years of patients’ follow-up (**Table 3**), we chose to combine these two parameters in order to assess their impact on survival rates. We confirmed high value of MIX score as risk factor for OS, regardless of pTNM staging (**Figure 4**).

Survival rates of MIX-high staining associated with late-stage patients are significantly poorer compared to MIX-low staining with a doubling HR, observed at 5-years and at 7-years of OS (**Figure 4**). The same tendency was observed for the early stage patients, even if only results acquired at 5-years of OS were significant (**Figure 4**).

Noteworthy that results are statistically more pronounced and relevant for 5-years follow-up: patients characterized by a MIX-Low staining have higher survival rates than MIX-High patients (**Figure 4A, C**). To accurately estimate the prognosis value of MIX staining according to given stages, survival analysis (Kaplan-Meier and univariate Cox regression) according to Low or High MIX subpopulations at early (I/II) or late (III/IV) stages, were performed (**Figure S4**). At 5 years of patients’ follow-up, a MIX-High staining could be considered as a poor prognosis marker in both early (HR: 3.3 with 95% CI 1.2 to 9.1, p=0.021; **Figure S4A**) and late stages (HR: 2.2 with 95% CI 1 to 4.6, p=0.039; **Figure S4B**). At 7 years of patients’ follow-up, MIX prognosis benefit is lost for early stages (**Figure S4C**) but is slightly maintained for late stages (HR: 1.95 with 95% CI 0.93 to 4.1, p=0.076; **Figure S4D**). Altogether, these results suggest that the MIX could be considered as an efficient prognosis marker to predict disease aggressiveness from early phase and within the 5 years post resection. Importantly, the significance of MIX prognosis value should be useful at early stage to adapt therapeutic strategy and improve patients’ management.

Thus, the cohort of early stage subpopulation (initially made up of 16 stage 1 and 25 stages 2; **Table S1**) was implemented (**Table S3**) with a total of 41 patients of stage I and 29 patients of stage II, at 5 years of follow-up. Survival analysis performed on this cohort confirms the High-MIX staining as poor prognosis factor. Although a slightly difference is observed between MIX-high and -Low in early stage subpopulation (p=0.18), it can be noted that MIX-high has a moderate bad prognosis value in univariate Cox model (p=0.18, HR=1.764 and 95% CI 0.6514-4.779; data not shown). Thus, survival rates of I and II early stages were analyzed separately, by combining pTNM stages and MIX scoring (*i.e.*, Stage I/MIX-Low, Stage I/MIX-High, Stage II/MIX-Low, Stage II/MIX-High; **Figure S5**). Interestingly, survival rates of Stage II/MIX High patients are collapsed compared to Stage I/MIX High, suggesting that the relative risk is markedly increased when a High MIX staining is detected in stage II patients.

To resume, if we consider 7 years of OS, the ColoSTEM Dx kit does not allows prediction of disease evolution (i.e., patients’ prognosis) regardless of their age or sex. However, concerning their stages, prognostic value of MIX staining appeared more reliable in the later stages of colon cancer patients (**Figure S4C, D**). On the contrary, if we consider 5 years of overall survival, the ColoSTEM Dx kit markedly predicts disease aggressiveness and allows the stratification of patients with good or poor prognosis, with a high or low risk of relapse after curative surgery, especially from early stages. Altogether, these results evidence that specific glycan motif of colon CSCs detected by the ColoSTEM Dx kit, constitute independent prognosis factor from pTNM staging and other clinicopathological data. It allows to discriminate a better or worse prognosis, as well as to predict in a standardized way colon cancer aggressiveness within the first 5 years after curative surgery.

### The ColoSTEM Dx Kit Displays Great Clinical Performances

Clinical performances of the ColoSTEM Dx kit, *i.e*., diagnostic specificity and sensitivity, have been determined from N=166 tumor tissues (N=86 from AMSBIO TMA and N=80 from the CRB-ICM Montpellier cohort) and N=136 tumor edges (N=86 from AMSBIO TMA and N=50 from the CRB Montpellier cohort). Among the N=166 tumor tissues, N=36 depicted an absence of MIX staining (false negatives) and N=130 were stained (true positives). Among the N=136 non tumor tissues, N=96 depicted an absence of MIX staining (true negatives) and N=40 were stained (false positives). According to formula described in “*Materials and Methods*” section, diagnostic sensitivity and specificity reach respectively 78.3% and 70.6%. ROC curve built from results of MIX staining on non-tumor and tumor colon tissue permit to establish that AUC is acceptable (AUC = 0.7445; data not shown) and confirmed a good sensitivity and specificity of ColoSTEM Dx kit.

To evaluate the cellular type specificity of ColoSTEM Dx kit, we have performed MIX staining on 18 kidney tumors samples. Three kidney tumors were positive and 15 tumors were negative. Compared to colorectal tumors, where 130 were positive and 36 were negative after MIX staining, we can conclude that there is a positive association between MIX staining and colorectal tumors (confirmed by Fisher’s exact test, p = 3.3x10-7).

### Specific Glycan Motifs of Colon CSCs Evidenced by the ColoSTEM Dx Kit Could Also Constitute Promising Predictive Biomarkers

**Figure 5:**
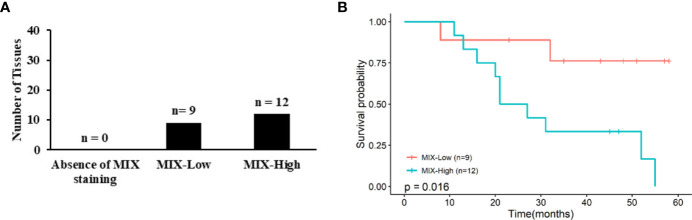
Association between MIX scoring with chemotherapeutic-treated patients’ survival rates at 5-years. **(A)** Graph represent numbers (N) of tumor samples from treated patients for which there is an absence of MIX, a MIX-Low or a MIX-High staining. All samples included (N=21) were stained with the MIX. **(B)** Corresponding Kaplan-Meier curves are displayed according to MIX staining (i.e., Mix-Low and -High). P values correspond to log-rank test.

In order to evaluate predictive values of the ColoSTEM Dx kit, 21 tumor tissues from chemotherapeutic-treated patients were stained with the MIX: 42.8% and 57.1% tissues were respectively MIX-Low and MIX-High (**Table S2** and **Figure 5A**). Kaplan Meier curves were achieved with treated patients’ OS rates at 5-years according to MIX staining levels (Low *vs* High; **Figure 5B**). 5-years OS of the MIX-High subgroup is significantly poorer than the MIX-Low, with a strong decrease in survival median (p=0.016). Univariate Cox analysis revealed MIX score (p=0.03) and, to a lesser extent, age (p=0.066) and pTNM staging (p=012), as predictive factor for OS in treated patients (**Table 4**, left panel). Multivariate analysis confirm that MIX score is an independent prognostic factor (HR: 6.98 with 95%CI 1.1 to 44.03, p=0.0387, **Table 4** right panel).

**Table 4:** Prognostic values of clinicopathological features (sex, age, stage and MIX staining) at 5 years of patients’ follow-ups (60 months) of chemotherapeutic-treated patients’ cohort.

		Univariate Cox analysis			Multivariate Cox analysis		
		HR	95% CI of HR	P Value	HR	95% CI of HR	P Value
**Sex**	**Female (ref.)**						
	**Male**	2.01	0.65 - 6.26	0.229	/	/	/
**Age**	**< 60 yrs (ref.)**						
	**> 60 yrs**	7.01	0.88 - 56.06	**0.067**	15.48	1.52 - 158.16	**0.021**
**Stage (UICC)**	**Early (I/II) (ref.)**						
	**Late (III/IV)**	2.91	0.76 - 11.19	0.12	4.23	0.94 - 18.98	0.0596
**MIX staining score**	**MIX-Low (ref.)**						
	**MIX-High**	5.42	1.17 - 25.07	**0.031**	6.98	1.11 - 44.03	**0.039**

Univariate and multivariate Cox regression were displayed for analysis of clinicopathological parameters and MIX scoring impact on prognostic value. CI, Confidence Interval; HR, Hazard Ratio; OS, Overall Survival; UICC, Union for International Cancer Control; yrs, years.

The p values less than or equal to 0.05 have been written in bold.

These preliminary results evidence that the ColoSTEM Dx kit might also constitute a promising predictive tool, i.e., companion test, in order to (i) allow better prediction of therapeutic responses and relapses’ risk and (ii) improve therapeutic management for each patient.

## Discussion

CSCs play a key role in colon cancer evolution and has major implications to cancer therapy. Currently, CSCs failed to be used as biomarkers in clinical routine although these cells could reflect tumor aggressiveness and might be of prime importance for diagnosis/prognosis. Indeed, CSCs enable cancer therapeutic resistance to conventional treatments thereby conduce to therapeutic failure. Thus, the reliable detection of CSCs from patient samples might improve future patient management and survival. Nevertheless, no kits or devices developed for clinical or translational research are currently likely to specifically and efficiently detect CSCs within tissues. Furthermore, the clinical use and significance of CSCs biomarkers are still restricted due to the risk of confusing detection with biomarkers expressed by adult non-cancerous stem cells as well as differentiated cancer cells (10, 12, 19).

In this context, Carcidiag Biotechnologies decided to develop a new device for a specific detection of CSCs and tumor cells related to CSCs in FFPE tissues from patient solid biopsies, usable in a clinic-standardized way in order to improve patients’ diagnosis and prognosis. ColoSTEM Dx kit, developed by Carcidiag Biotechnologies, is based on glycoproteins detection, known to be specifically (over)expressed at the surface of colon CSCs and tumor cells related to CSCs. More precisely, this diagnosis tool uses a MIX of biotinylated plant lectins that recognize glycan patterns specifically expressed or overexpressed by colon CSCs only, *i.e*, normal SCs or differentiated tumor cells are not detected by the lectin MIX. Strikingly, the MIX staining score in stained cells is higher suggesting a strong percentage of CSC. While the number of CSCs in colon cancer is generally reduced to a low percentage of total cells, this difference might be due to cell plasticity that contributes to increase the percentage of CSCs or tumor cells related to CSCs. Indeed, non-stem tumor cells can emphasize an oncogenic transformation enhancing their spontaneous conversion in cancer stem cell (CSCs)-like cells (36). This interconversion also occurs *in vivo*, CSCs-depleted fractions might give rise to tumors enriched in CSCs or cancer stem-like cells (37). A second mechanism combined to previous might explain the significant rise of MIX positive cells observed in score 3 (**Figure 1**) and thus the increase of CSCs number detected by ColoSTEM Dx kit. CSCs or tumor cells related to CSCs are likely to transfer their aggressiveness properties or stemness phenotypes to recipient non-CSCs *via* the dissemination of extracellular vesicles triggering their transformation in tumor cells with acquired stem-like properties (38, 39). Altogether, these data suggest the MIX staining might not detect only CSCs but also the colon cancer cells which gained some of “stemness” properties and are considered as cancer stem-like cells.

Here, it was evidenced that MIX-positive cells from HT-29 cell sorting show an enrichment of EpCAM^high^ and ALDH1^high^ cell subpopulations, consistent with a stem cell phenotype. Indeed, EpCAM expression and ALDH1 activity are both currently used to define CSCs populations in digestive cancers (40). In an experimental context, their expressions were associated with poor prognosis in both disease-free and overall survival for colorectal cancer. However, these markers are not adaptable to clinical routine use. In addition, MIX positive cells are highly able to form colonospheres, up to 8 times more than their negative counterpart, again reflecting the stem cell status of these cells. Based on this *in vitro* evaluation, the potential stem cell detection capacity of MIX was tested by IHC on 42 colorectal cancer tissue samples and the score obtained with the MIX staining (Low *vs* High) was compared to that of OCT-4 staining, a common SCs marker. While MIX-high staining seems restricted to a subset of tissues, OCT4-high staining is present in a broad panel of tissues. No significant association was been evidenced between intensity of staining with both markers. These results are consistent with previous work demonstrating that although OCT-4 is considered as a pluripotent SCs marker required to enhance the self-renewal ability, its expression was reported to be restricted in normal colon, polyp and colon cancer. Thus, OCT-4 analysis by IHC is poor of interest to characterize CSCs for diagnosis (41). Survival curves according low or high OCT-4 staining were homogeneous, while MIX-high staining reveals a significant decrease of OS, suggesting that this biomarker is clearly more relevant for monitoring patients and might be of prime interest for patient management. Consistently, it appeared that MIX-High staining is considered as an independent bad prognosis factor as validated by univariate and multivariate Cox regression analysis (HR: 4.2). Poor prognosis value was confirmed by the decreased median survival of colon cancer patients characterized by an OCT4-High/MIX-High co-staining. As previously mentioned, we confirmed that OCT-4 staining alone (High *vs* Low) is unable to discriminate good or poor prognosis patients. These results suggest that MIX staining allows to recognize CSCs and tumor cells related to CSCs and could be useful for their detection in tumor samples and thus could predict the presence of CSCs and tumor cells related to CSCs as well as the associated risk of recurrence post-resection.

In this context, the prognosis significance of the ColoSTEM Dx kit has been assessed on several cohorts of non-chemotherapeutic-treated (total of N=208 tissues) and chemotherapeutic-treated (total of N=21 tissues) colon cancer patients, according to MIX scoring and clinicopathological data available (gender, age and stages). The MIX staining has revealed a significant prognosis value especially at early stages within the 5 years of patient follow-up, independently of pTNM staging. The prognosis value of MIX staining on OS at 5 years was confirmed but not demonstrated at 7 years suggesting that the MIX could be of prime importance at early stages of colon cancer and might be used to predict treatment outcome at this stage. Since the MIX staining constitute an independent poor prognosis factor, from pTNM staging and from other clinical parameters (age and gender), it could be crucial in the future management of patients. In a similar way, patients stratification using combination of MIX and pTNM stages allows improvement of the patient’s classification. Indeed, this combined analysis highlights that MIX-High staining is a marker of poor prognosis and could be used as a predictive biomarker to improve management of patients. Indeed, regardless of stage, MIX-high is associated with a poor prognosis with a HR of 3 to 8 in early or late stage, respectively, at 5 years of follow-up. The same trend was observed at 7 years, without significance. Thus, patients who are still alive at 7 years in the OS cohort are probably very good prognosis colorectal cancer. The bad prognosis value of MIX staining has been confirmed in chemotherapeutic-treated patients’ cohort, supporting that the MIX could also be likely to predict patients’ treatment outcome at early stages and maybe in future to prevent tumor burden by early detection of recurrence.

Even if new tools such as Immunoscore and circulating tumor DNA aid to accurately characterize patients with minimal residual disease, they don’t allow to identify the specific presence of CSCs or tumor cells related to CSCs within tumor. The presence of circulating cancer cells does not predict the presence of CSCs within the tumor mass; currently, no direct relationship between the presence of circulating cancer cells and the presence of CSCs was been found in the literature. On the contrary, the ColoSTEM Dx kit is efficient to detect CSCs and cancer stem-like cells even from early-stage tumor. It can therefore be complementary to current approaches. Nevertheless, further developments are required and will include validation in prospective multicenter interventional outcome studies in order to confirm on a wide cohort of chemotherapeutic-treated patients that MIX staining could have a predictive value in the early stages. This newly prognostic tool could be spread to any kind of solid cancer [39] and it appear very promising as there is currently no kit used in clinical routine for the detection of CSC and cancer stem-like cells to our knowledge.

In summary, it appears that ColoSTEM Dx kit demonstrated its significance to detect CSCs or tumor cells related to CSCs, more efficiently than OCT-4 and could be a new tool usefully in clinical management of colon cancer, due to their potential to predict tumor aggressiveness, even on colorectal cancer early stages.

## Institutional Review Board Statement

The study was conducted according to the guidelines of the Declaration of Helsinki, and approved by the Institutional Review Board (or Ethics Committee) of Limoges University Hospital. Tumor samples were provided by Biological Resources Collection (BRC-Biolim-Cancer) from Limoges University Hospital certified NF S 96900 since 2014. Reporting number of collections held by the BRC-Biolim-Cancer: DC-2010-1074. Authorization number for the transfer of biological resources: AC-2013-1853.

## Patents

The patent under the national registration number 1561763 (N° WO2016FR53196 and WO2016FR53197 publication number 3044680 and 3044681) results from a part of the work reported in this manuscript, i.e., experimental data depicted in the supplementary materials (**Figure S1**).

## Data Availability Statement

The original contributions presented in the study are included in the article/**Supplementary Material**. Further inquiries can be directed to the corresponding authors.

## Ethics Statement

The study was conducted according to the guidelines of the Declaration of Helsinki, and approved by the Institutional Review Board (or Ethics Committee) of Limoges University Hospital. Tumor samples were provided by Biological Resources Collection (BRC-Biolim-Cancer) from Limoges University Hospital certified NF S 96900 since 2014. Reporting number of collections held by the BRC-Biolim-Cancer: DC-2010-1074. Authorization number for the transfer of biological resources: AC-2013-1853. The patients/participants provided their written informed consent to participate in this study.

## Author Contributions

Conceptualization, SD, FL, M-OJ, VC and MM. Methodology, CB, MP, AL, SeB, SD, FL, M-OJ and MM. Validation, AL, SaB, SD, FL, M-OJ, VC and MM. Formal analysis, AL, CB, SaB, SD, FL. Investigation, FL, M-OJ and MM. Resources, AC, FL, M-OJ and MM. Data curation, AC and MM. Writing-original draft preparation, SaB, SD, NC, VC, FL and MM. Writing-review and editing, SaB, SD, VC, FL and MM. Visualization, SaB, SD, FL, M-OJ, VC and MM. Supervision, FL, M-OJ, VC and MM. Project administration, FL, M-OJ, VC and MM. Funding acquisition, FL, M-OJ, VC and MM. All authors have read and agreed to the published version of the manuscript.

## Funding

This research was funded by SATT Grand-Centre, grant number 0015-UNILIM-DIALECT.

## Conflict of Interest

Authors SB, CB, MP, and VC were employed by Carcidiag Biotechnologies company.

The remaining authors declare that the research was conducted in the absence of any commercial or financial relationships that could be construed as a potential conflict of interest.

## Publisher’s Note

All claims expressed in this article are solely those of the authors and do not necessarily represent those of their affiliated organizations, or those of the publisher, the editors and the reviewers. Any product that may be evaluated in this article, or claim that may be made by its manufacturer, is not guaranteed or endorsed by the publisher.
