# Supplementary material for: Detection of Glycosylated Markers From Cancer Stem Cells With ColoSTEM Dx Kit for Earlier Prediction of Colon Cancer Aggressiveness
Source: Front Oncol. 2022 Jul 22;12:918702. doi: 10.3389/fonc.2022.918702 (PMC9355573; doi:10.3389/fonc.2022.918702)
Supplement: Supplementary file 1 [file DataSheet_1.docx]

Supplementary Material

# Supplementary Figures and Tables

## Supplementary Figures

Supplementary Figure 1. *In vitro* characterization of MIX (ColoSTEM Dx kit) efficiency in colon CSCs detection and enrichment. **(A)** Histogram representing EpCAM^High^ cell percentages *ratio* analyzed by FCM within AC133+ and MIX+ sorted-cell, compared to negative sorted-cells normalized to 1 (dotted line). **(B)** Cytogram representing ALDH1^bright^ cell percentages within MIX- and MIX+ sorted-cells. **(C)** Evaluation of sphere forming ability from MIX- and MIX+ sorted-cells, according to seeded cell densities (600, 1250, 2500, 5000 and 10000 cells/well). Representative illustrations are depicted at left panel (magnification, x100). Histograms (right panel) represent the mean of sphere number formed from either MIX- or MIX+ sorted-cells **P<0.05, **P<0.01, ***P<0.001* *ALDH1: Aldehyde dehydrogenase 1; EpCAM: Epithelial Cell Adhesion Molecule; FCM: Flow Cytometry; +: positive; -: negative.*

**Supplementary Figure 2.** Distribution of tumor tissues from non-treated patients according to MIX, OCT-4 or MIX/OCT-4 staining. Distribution of tumor tissues from non-treated patients for which there is (A) an absence of MIX, a MIX-Low or a MIX-High staining, (B) an absence of OCT-4, an OCT-4-Low or an OCT-4-High staining, and (C) a MIX-Low/OCT-4-Low, a MIX-Low/OCT-4-High, a MIX-High/OCT-4-Low, or a MIX-High/OCT-4-High co-staining. All tissues included (N=42) have been stained.

**Supplementary Figure 3.** Association between gender and age with survival rates at 5- and 7 years (60 and 84 months respectively). Prognostic value of each clinical characteristic was assessed using the Kaplan-Meier method and log-rank test by stratification of patients according to either female or male gender or age class, with a cutoff of 60 years at 5-years of OS **(A)** and at 7-years of OS **(B)***. OS: Overall survival; yrs: years.*

**Supplementary Figure 4.** Association between MIX-staining with survival rates at 5- and 7 years (60 and 84 months respectively), according to either early stages (I/II) **(A and C)** or late stages (III/IV) **(B and D)**. Corresponding Kaplan-Meier curves are displayed according to MIX staining (i.e., Mix-Low and -High). *P* values correspond to log-rank test. Prognostic value of MIX staining associated to early or late stages, was assessed using univariate Cox model*. CI: Confidence Interval; HR: Hazard Ratio; yrs: years.*


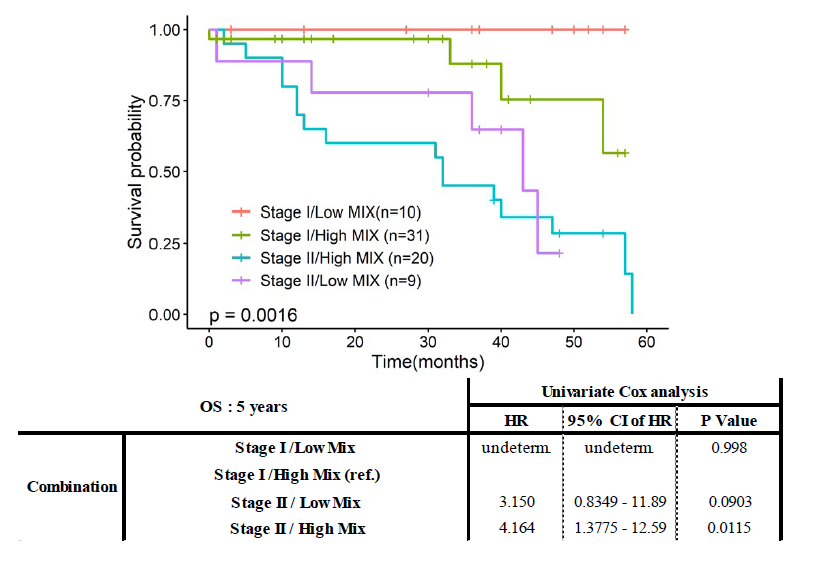


**Supplementary Figure 5.** Combination of pTNM staging (early stages, i.e., I and II) and MIX scoring for survival analysis at 5-years (60 months). Comparison of survival curves was performed using Kaplan-Meier method (with log-rank test) by stratification of 70 patients (Table S3) according to stages I and II or Low- and High-MIX scoring, at 5-years **(A)**. Prognostic values of early stages and MIX scoring for survival analysis at 5-years of patients’ follow-up were analyzed and resulting HR (with 95% CI) for stratifying patients for 5-years of OS according to stages and MIX scoring combination, were obtained by univariate Cox modeling **(B)**. *CI: Confidence Interval; HR: Hazard Ratio; OS: Overall Survival; pTNM: pathology Tumor Node Metastasis.*

## Supplementary Tables

**Supplementary Table 1.** Characteristics of non-chemotherapeutic-treated patients with colon cancer (N=128, all stages) and corresponding MIX and OCT-4 scoring. In blue are mentioned patients from Limoges’ Hospital included for MIX/OCT-4 co-staining (N=42). In green are mentioned patients from the commercial TMA (HCol-Ade180Sur-08, AMSBIO) (N=86). In italic are mentioned patients, among the N=128, that were included for 7-years survival rates analysis (≤ 84 months; N=74). M: Men; TMA: Tissue microarray; W: Women; 0: living; 1: dead.

| **Patients’coding** | **Stage** | **Gender** | **Age** | **State (Living or dead)** | **Survival (months)** | **MIX scoring** | **OCT-4 scoring** |
| --- | --- | --- | --- | --- | --- | --- | --- |
| **11000749** | II | M | 81 | 0 | 37 | 2 | 3 |
| **11000760** | III | M | 79 | 1 | 14 | 1 | 2 |
| **11002340** | III | W | 86 | 1 | 49 | 1 | 1 |
| **11002384** | III | M | 87 | 1 | 0 | 3 | 1 |
| **11003103** | IV | W | 87 | 1 | 3 | 3 | 3 |
| **11003115** | III | M | 80 | 1 | 51 | 3 | 3 |
| **11003171** | I | W | 86 | 0 | 54 | 2 | 3 |
| **11003299** | III | W | 81 | 0 | 58 | 2 | 2 |
| **11003732** | I | W | 82 | 0 | 57 | 2 | 3 |
| **11003807** | II | M | 97 | 1 | 2 | 3 | 3 |
| **11006429** | I | M | 61 | 0 | 37 | 1 | 2 |
| **11006692** | IV | M | 59 | 1 | 0 | 3 | 3 |
| **11006768** | II | W | 85 | 1 | 1 | 1 | 1 |
| **11006839** | III | M | 80 | 0 | 37 | 1 | 3 |
| **11007233** | II | W | 86 | 1 | 45 | 2 | 3 |
| **11008214** | II | M | 67 | 0 | 54 | 3 | 3 |
| **11009216** | II | M | 55 | 0 | 36 | 0 | 2 |
| **11009928** | II | M | 80 | 1 | 10 | 3 | 3 |
| **11009993** | I | W | 80 | 0 | 52 | 2 | 2 |
| **11010218** | I | M | 85 | 0 | 41 | 3 | 3 |
| **11010373** | I | W | 60 | 0 | 50 | 2 | 3 |
| **11011091** | I | W | 71 | 0 | 47 | 1 | 3 |
| **11011399** | IV | M | 81 | 1 | 8 | 3 | 3 |
| **11011957** | III | M | 85 | 1 | 26 | 3 | 3 |
| **11012863** | II | M | 69 | 0 | 48 | 2 | 3 |
| **11A00831** | II | W | 50 | 0 | 48 | 3 | 3 |
| **11A00858** | II | W | 88 | 1 | 12 | 3 | 3 |
| **11A04294** | I | M | 85 | 1 | 0 | 3 | 3 |
| **11A04525** | I | M | 70 | 0 | 30 | 3 | 3 |
| **11A04576** | III | W | 80 | 0 | 48 | 3 | 3 |
| **11A04734** | II | W | 52 | 0 | 30 | 2 | 3 |
| **11A05140** | II | M | 65 | 0 | 40 | 2 | 3 |
| **12A01654** | I | W | 85 | 1 | 40 | 3 | 3 |
| **12A04757** | I | W | 64 | 0 | 38 | 3 | 1 |
| **12A05219** | I | M | 84 | 0 | 32 | 3 | 3 |
| **12A05717** | IV | M | 70 | 1 | 0 | 3 | 3 |
| **12A06343** | I | M | 68 | 0 | 27 | 2 | 3 |
| **12A10391** | I | M | 78 | 0 | 36 | 3 | 3 |
| **12A12598** | IV | W | 56 | 1 | 0 | 2 | 3 |
| **12A14677** | IV | M | 70 | 1 | 1 | 3 | 1 |
| **12A15699** | I | M | 64 | 0 | 38 | 3 | 3 |
| **12A18403** | I | W | 77 | 0 | 36 | 2 | 3 |
| A1 | II | W | 78 | 1 | 32 | 3 | NA |
| A11 | III | M | 57 | 1 | 10 | 0 | NA |
| *A13* | *II* | *M* | *76* | *0* | *83* | *1* | *NA* |
| *A15* | *III* | *W* | *60* | *0* | *83* | *3* | *NA* |
| A17 | II | W | 60 | 1 | 40 | 3 | NA |
| A5 | II | W | 81 | 1 | 14 | 1 | NA |
| *A7* | *II* | *W* | *64* | *0* | *83* | *3* | *NA* |
| *A9* | *II* | *M* | *62* | *0* | *83* | *0* | *NA* |
| *B1* | *III* | *M* | *61* | *0* | *83* | *0* | *NA* |
| *B11* | *II* | *W* | *70* | *0* | *82* | *1* | *NA* |
| *B13* | *II* | *W* | *76* | *0* | *82* | *3* | *NA* |
| B15 | II | M | 57 | 1 | 43 | 1 | NA |
| B3 | II | W | 54 | 1 | 9 | 0 | NA |
| *B5* | *III* | *M* | *53* | *0* | *83* | *2* | *NA* |
| *B7* | *II* | *M* | *74* | *0* | *83* | *3* | *NA* |
| B9 | II | M | 74 | 1 | 47 | 3 | NA |
| *C1* | *II* | *M* | *65* | *0* | *82* | *1* | *NA* |
| *C11* | *III* | *W* | *47* | *0* | *81* | *3* | *NA* |
| *C13* | *II* | *M* | *68* | *0* | *81* | *3* | *NA* |
| *C15* | *II* | *W* | *72* | *0* | *81* | *3* | *NA* |
| C17 | III | M | 90 | 1 | 8 | 3 | NA |
| C3 | III | W | 50 | 1 | 27 | 1 | NA |
| *C5* | *II* | *M* | *80* | *0* | *82* | *3* | *NA* |
| C7 | III | W | 70 | 1 | 2 | 3 | NA |
| C9 | III | W | 73 | 1 | 28 | 1 | NA |
| *D1* | *III* | *M* | *65* | *0* | *81* | *1* | *NA* |
| *D11* | *II* | *W* | *55* | *0* | *80* | *3* | *NA* |
| *D13* | *I* | *M* | *68* | *0* | *80* | *3* | *NA* |
| D15 | III | M | 76 | 1 | 10 | 1 | NA |
| *D17* | *III* | *M* | *85* | *0* | *80* | *3* | *NA* |
| *D3* | *II* | *M* | *61* | *0* | *81* | *3* | *NA* |
| D5 | III | W | 61 | 1 | 10 | 3 | NA |
| D7 | II | W | 78 | 1 | 16 | 3 | NA |
| D9 | II | M | 67 | 1 | 39 | 3 | NA |
| *E1* | *II* | *W* | *63* | *0* | *80* | *3* | *NA* |
| E11 | III | M | 62 | 1 | 16 | 3 | NA |
| *E13* | *II* | *M* | *75* | *0* | *79* | *3* | *NA* |
| *E15* | *III* | *M* | *58* | *0* | *79* | *2* | *NA* |
| E17 | II | W | 83 | 1 | 5 | 3 | NA |
| *E3* | *II* | *M* | *62* | *0* | *80* | *3* | *NA* |
| E5 | III | W | 72 | 1 | 9 | 3 | NA |
| *E7* | *II* | *W* | *78* | *0* | *80* | *3* | *NA* |
| *E9* | *II* | *M* | *63* | *0* | *80* | *1* | *NA* |
| *F1* | *II* | *M* | *75* | *0* | *78* | *3* | *NA* |
| F11 | III | M | 65 | 1 | 29 | 1 | NA |
| F13 | III | M | 80 | 1 | 11 | 3 | NA |
| *F15* | *II* | *W* | *75* | *0* | *77* | *3* | *NA* |
| F17 | II | W | 60 | 1 | 31 | 3 | NA |
| *F3* | *I* | *M* | *70* | *1* | *75* | *3* | *NA* |
| *F5* | *II* | *M* | *51* | *0* | *78* | *1* | *NA* |
| *F7* | *II* | *W* | *86* | *0* | *78* | *3* | *NA* |
| F9 | III | M | 72 | 1 | 44 | 3 | NA |
| *G1* | *II* | *M* | *78* | *0* | *77* | *3* | *NA* |
| *G13* | *II* | *W* | *61* | *0* | *77* | *1* | *NA* |
| G15 | III | M | 73 | 1 | 32 | 1 | NA |
| *G17* | *III* | *M* | *71* | *0* | *76* | *2* | *NA* |
| *G3* | *III* | *M* | *51* | *0* | *77* | *3* | *NA* |
| *G5* | *I* | *M* | *72* | *0* | *77* | *3* | *NA* |
| G7 | II | W | 72 | 1 | 13 | 3 | NA |
| G9 | II | M | 65 | 1 | 12 | 3 | NA |
| H1 | III | M | 65 | 1 | 13 | 3 | NA |
| H11 | III | M | 60 | 1 | 22 | 1 | NA |
| *H13* | *II* | *W* | *69* | *0* | *75* | *3* | *NA* |
| *H15* | *II* | *W* | *64* | *0* | *74* | *3* | *NA* |
| H17 | II | M | 65 | 1 | 38 | 0 | NA |
| H3 | III | W | 52 | 1 | 16 | 1 | NA |
| *H5* | *I* | *W* | *81* | *0* | *76* | *3* | *NA* |
| H7 | II | W | 67 | 1 | 36 | 2 | NA |
| H9 | III | M | 54 | 1 | 45 | 1 | NA |
| I1 | III | M | 83 | 1 | 18 | 1 | NA |
| *I11* | *III* | *W* | *54* | *0* | *74* | *1* | *NA* |
| *I13* | *II* | *W* | *62* | *1* | *66* | *0* | *NA* |
| *I15* | *III* | *M* | *74* | *0* | *74* | *1* | *NA* |
| *I17* | *I* | *M* | *67* | *0* | *74* | *3* | *NA* |
| *I3* | *I* | *W* | *54* | *0* | *74* | *1* | *NA* |
| *I5* | *II* | *W* | *79* | *0* | *74* | *3* | *NA* |
| *I7* | *II* | *W* | *60* | *0* | *74* | *0* | *NA* |
| *I9* | *III* | *W* | *50* | *0* | *74* | *0* | *NA* |
| J1 | III | W | 65 | 1 | 36 | 0 | NA |
| J11 | II | W | 71 | 1 | 32 | 3 | NA |
| J13 | II | W | 75 | 1 | 57 | 3 | NA |
| J15 | IV | W | 70 | 1 | 51 | 1 | NA |
| J17 | III | W | 76 | 1 | 4 | 0 | NA |
| *J3* | *III* | *M* | *75* | *0* | *74* | *0* | *NA* |
| *J5* | *II* | *M* | *70* | *0* | *74* | *0* | *NA* |
| *J7* | *II* | *W* | *73* | *0* | *74* | *3* | *NA* |

**Supplementary Table 2.** Characteristics of chemotherapeutic-treated patients with colon cancer (N=21) and corresponding MIX scoring. Patients were collected from Limoges’ Hospital. M: Men; W: Women; 0: living; 1: dead.

| **Patients’coding** | **Stage** | **Gender** | **Age** | **State (Living or dead)** | **Survival (months)** | **MIX scoring** |
| --- | --- | --- | --- | --- | --- | --- |
| 11A01990 | 2 | M | 77 | 0 | 48 | 1 |
| 11002915 | 3 | W | 81 | 0 | 57 | 1 |
| 11008866 | 1 | W | 78 | 0 | 43 | 2 |
| 11004010 | 2 | W | 82 | 0 | 23 | 2 |
| 11008107 | 2 | M | 61 | 1 | 32 | 2 |
| 11009896 | 2 | W | 56 | 0 | 35 | 2 |
| 11008733 | 2 | W | 37 | 0 | 51 | 2 |
| 11011448 | 3 | W | 79 | 1 | 8 | 2 |
| 11001257 | 3 | W | 53 | 0 | 58 | 2 |
| 11008177 | 2 | M | 63 | 1 | 27 | 3 |
| 11003280 | 2 | W | 60 | 0 | 45 | 3 |
| 11005318 | 2 | W | 60 | 1 | 52 | 3 |
| 11001594 | 4 | M | 62 | 1 | 11 | 3 |
| 12A06313 | 4 | W | 76 | 1 | 13 | 3 |
| 12A04358 | 4 | W | 77 | 1 | 16 | 3 |
| 12A06813 | 4 | W | 72 | 1 | 20 | 3 |
| 11A02607 | 4 | W | 63 | 1 | 21 | 3 |
| 11013132 | 4 | W | 35 | 0 | 47 | 3 |
| 11005184 | 4 | M | 59 | 1 | 55 | 3 |
| 12A8880 | 4 | M | 62 | 1 | 21 | 3 |
| 12A15069 | 4 | M | 69 | 1 | 31 | 3 |
|  |  |  |  |  |  |  |

**Supplementary Table 3.** Characteristics of non-chemotherapeutic-treated early stages (I and II) patients with colon cancer (N=70) and corresponding MIX scoring. Patients from Montpellier’ CRB (n=29), Limoges’ Hospital (n=27) and commercial TMA (n=14) are mentioned in red, blue and green, respectively. 0: living; 1: dead.

| **Patients’ coding** | **Stage** | **Survival (Months)** | **State (Living or dead)** | **MIX scoring** |
| --- | --- | --- | --- | --- |
| 0000001 | I | 1 | 0 | 3 |
| 0000002 | I | 54 | 1 | 3 |
| 0000003 | I | 1 | 0 | 3 |
| 0000004 | I | 28 | 0 | 3 |
| 0000009 | I | 1 | 0 | 3 |
| 0000011 | I | 33 | 1 | 3 |
| 0000013 | I | 13 | 0 | 3 |
| 0000018 | I | 44 | 0 | 3 |
| 0000019 | I | 3 | 0 | 3 |
| 0000021 | I | 3 | 0 | 3 |
| 0000024 | I | 9 | 0 | 3 |
| 0000025 | I | 3 | 0 | 3 |
| 0000026 | II | 48 | 0 | 3 |
| 0000028 | I | 14 | 0 | 3 |
| 0000029 | I | 32 | 0 | 3 |
| 0000030 | II | 58 | 1 | 3 |
| 0000033 | II | 10 | 1 | 3 |
| 0000034 | I | 10 | 0 | 3 |
| 0000035 | I | 2 | 0 | 3 |
| 0000039 | I | 57 | 0 | 3 |
| 0000039 | II | 39 | 0 | 3 |
| 0000040 | I | 3 | 0 | 1 |
| 0000041 | I | 13 | 0 | 1 |
| 0000042 | I | 2 | 0 | 3 |
| 0000043 | I | 2 | 0 | 3 |
| 0000045 | I | 17 | 0 | 3 |
| 0000047 | I | 3 | 0 | 3 |
| 0000048 | I | 57 | 0 | 3 |
| 0000049 | I | 56 | 0 | 3 |
| **11000749** | II | 37 | 0 | **2** |
| **11003171** | I | 54 | 0 | **2** |
| **11003732** | I | 57 | 0 | **2** |
| **11003807** | II | 2 | 1 | **3** |
| **11006429** | I | 37 | 0 | **1** |
| **11006768** | II | 1 | 1 | **1** |
| **11007233** | II | 45 | 1 | **2** |
| **11008214** | II | 54 | 0 | **3** |
| **11009928** | II | 10 | 1 | **3** |
| **11009993** | I | 52 | 0 | **2** |
| **11010218** | I | 41 | 0 | **3** |
| **11010373** | I | 50 | 0 | **2** |
| **11011091** | I | 47 | 0 | **1** |
| **11012863** | II | 48 | 0 | **2** |
| **11A00831** | II | 48 | 0 | **3** |
| **11A00858** | II | 12 | 1 | **3** |
| **11A04294** | I | 0 | 1 | **3** |
| **11A04525** | I | 30 | 0 | **3** |
| **11A04734** | II | 30 | 0 | **2** |
| **11A05140** | II | 40 | 0 | **2** |
| **12A01654** | I | 40 | 1 | **3** |
| **12A04757** | I | 38 | 0 | **3** |
| **12A05219** | I | 32 | 0 | **3** |
| **12A06343** | I | 27 | 0 | **2** |
| **12A10391** | I | 36 | 0 | **3** |
| **12A15699** | I | 38 | 0 | **3** |
| **12A18403** | I | 36 | 0 | **2** |
| A1 | II | 32 | 1 | **3** |
| A17 | II | 40 | 1 | **3** |
| A5 | II | 14 | 1 | **1** |
| B15 | II | 43 | 1 | **1** |
| B9 | II | 47 | 1 | **3** |
| D7 | II | 16 | 1 | **3** |
| D9 | II | 39 | 1 | **3** |
| E17 | II | 5 | 1 | **3** |
| F17 | II | 31 | 1 | **3** |
| G7 | II | 13 | 1 | **3** |
| G9 | II | 12 | 1 | **3** |
| H7 | II | 36 | 1 | **2** |
| J11 | II | 32 | 1 | **3** |
| J13 | II | 57 | 1 | **3** |
